# Palliative Care Resource Utilization in Patients with Prion Disease

**DOI:** 10.1002/alz70858_102218

**Published:** 2025-12-26

**Authors:** Gregory S Day, Roaa Zayat, Yoav D Piura, Brian S Appleby, Maisha T Robinson

**Affiliations:** ^1^ Mayo Clinic in Florida, Jacksonville, FL, USA; ^2^ University Hospitals Cleveland Medical Center, Cleveland, OH, USA; ^3^ Case Western Reserve University School of Medicine, Cleveland, OH, USA

## Abstract

**Background:**

Prion diseases are universally fatal causes of rapid progressive dementia owing to the formation and spread of prions throughout the brain. Palliative care focuses on improving quality of life for patients with serious or advanced medical conditions. Timely access to palliative care is central to the care of patients with prion disease and other progressive neurodegenerative diseases. We sought to evaluate palliative care utilization and mode of delivery in patients with prion disease.

**Method:**

Clinical data were extracted from the electronic medical records of patients with definite or probable prion disease diagnosed and managed across the Mayo Clinic Enterprise from January 2012 to August 2023. We systematically evaluated the frequency of symptoms and signs likely to benefit from palliative care and considered the relationship between these features and palliative care resource utilization, method of delivery, and outcomes.

**Result:**

172 patients with symptomatic prion disease were identified. 165 (94.8%) patients experienced ≥1 symptom/sign likely to benefit from palliative care, yet only 113 (65.7%) patients accessed palliative care resources during the illness course (hospice care: 39.7%; palliative care consultation: 14.9% inpatient, 6.9% outpatient; both: 3.4%). Lack of referral by the diagnosing clinician was the most common reason (55.9%) patients did not receive palliative care services. Early and predominant language dysfunction, behavioral changes, constipation, and emergency department visits or hospital admissions were independently associated with greater odds of referral for palliative care services. Goals of care (i.e., living environment, nutritional and hospitalization plans) and advanced care plans (i.e., healthcare surrogates, living will, code status, hospice) were more likely to be documented in patients receiving palliative care consultations (88.6% and 93.2%, respectively) versus hospice care alone (7.8% and 14.5%, respectively; *p* <0.001).

**Conclusion:**

More than a third of patients with prion disease did not receive palliative care resources despite a high prevalence of symptoms/signs likely to benefit from palliative care. These findings highlight an opportunity to enhance the care of patients with prion disease by promoting early access to palliative care resources that may support symptom management, enhance caregiver support, and reinforce patient autonomy by clarifying goals of care and advance care planning.

